# The Brain Salience Network at the Intersection of Pain and Substance use Disorders: Insights from Functional Neuroimaging Research

**DOI:** 10.1007/s40429-024-00593-9

**Published:** 2024-07-29

**Authors:** Xinyi Li, Gabriel Kass, Corinde E. Wiers, Zhenhao Shi

**Affiliations:** Department of Psychiatry, University of Pennsylvania Perelman School of Medicine, Philadelphia, PA 19104 USA

**Keywords:** Salience Network, Physical pain, Social pain, Substance use Disorders, Neuroimaging

## Abstract

**Purpose of Review:**

The brain’s salience network (SN), primarily comprising the anterior insula and anterior cingulate cortex, plays a key role in detecting salient stimuli and processing physical and socioemotional pain (e.g., social rejection). Mounting evidence underscores an altered SN in the etiology and maintenance of substance use disorders (SUDs). This paper aims to synthesize recent functional neuroimaging research emphasizing the SN’s involvement in SUDs and physical/socioemotional pain and explore the therapeutic prospects of targeting the SN for SUD treatment.

**Recent Findings:**

The SN is repeatedly activated during the experience of both physical and socioemotional pain. Altered activation within the SN is associated with both SUDs and chronic pain conditions, characterized by aberrant activity and connectivity patterns as well as structural changes. Among individuals with SUDs, functional and structural alterations in the SN have been linked to abnormal salience attribution (e.g., heightened responsiveness to drug-related cues), impaired cognitive control (e.g., impulsivity), and compromised decision-making processes. The high prevalence of physical and socioemotional pain in the SUD population may further exacerbate SN alterations, thus contributing to hindered recovery progress and treatment failure. Interventions targeting the restoration of SN functioning, such as real-time functional MRI feedback, neuromodulation, and psychotherapeutic approaches, hold promise as innovative SUD treatments.

**Summary:**

The review highlights the significance of alterations in the structure and function of the SN as potential mechanisms underlying the co-occurrence of SUDs and physical/socioemotional pain. Future work that integrates neuroimaging with other research methodologies will provide novel insights into the mechanistic role of the SN in SUDs and inform the development of next-generation treatment modalities.

## Introduction

Allocating attentional resources wisely to relevant and important information is vital for overall adaptability in a constantly changing world. The brain’s salience network (SN), which primarily comprises the anterior insula (AI) and the anterior cingulate cortex (ACC), plays a pivotal role in facilitating information prioritization by directing attention to salient stimuli that are emotionally charged, novel, or otherwise behaviorally relevant or important for survival [1]. Notably, the SN is strongly implicated in the processing of pain – both physical [2, 3] and socioemotional [4, 5]. Individuals with substance use disorders (SUDs) exhibit structural and functional alterations in the salience network (SN) that are linked to various cognitive and behavioral deficits [6–8]. In this article, we review the growing evidence from functional neuroimaging research that highlights the SN in underlying the mutually reinforcing effects of SUDs and physical/socioemotional pain as well as the therapeutic prospects of targeting the SN for SUD treatment.

### The Brain Salience Network

**Fig. 1:**
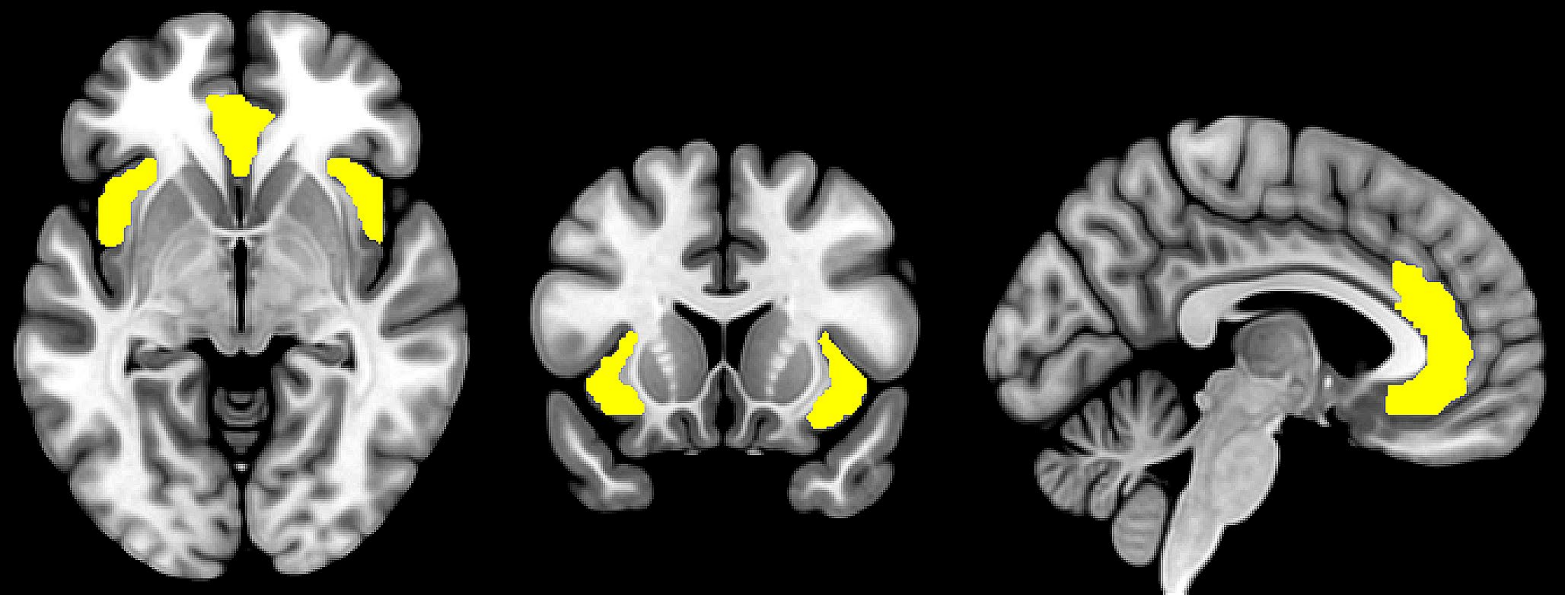
The anterior insula and anterior cingulate cortex (yellow) (Montreal Neurological Institute coordinates: x/y/z=–5/15/–5) defined in the Neuromorphometrics atlas (www.neuromorphometrics.com)

Studies of interregional brain connectivity using functional MRI have unveiled distinct large-scale networks of brain regions that support various cognitive processes. The SN is one of the most well-characterized brain networks and is involved in dynamically monitoring internal and external stimuli, directing attention toward salient and relevant information while filtering out distractions [1]. It is primarily anchored in two hub regions: the bilateral AI and the dorsal portion of the ACC [8–10] (see Fig. 1). Other brain regions, such as the ventrolateral prefrontal cortex, the inferior parietal lobule/temporoparietal junction, the thalamus, and the amygdala have also been considered components of the SN though with less clear consensus [8, 9]. While the exact anatomical boundaries of the SN are not well-defined, the reciprocal anatomical connections among the SN regions are assumed to underpin their coordinated neurocognitive functions [11, 12] that extend beyond mere sensory processing but also include affective processing, decision making, error monitoring, social cognition, interoceptive awareness, among others [1]. Stimuli of high perceived importance and relevance consistently activate brain regions in the SN, which guides the direction of attention and cognitive resources toward the process of decision-making and behavior selection [10, 13]. The SN closely interacts with other brain networks such as the default mode network (DMN), which is involved in internally directed tasks (e.g. self-reflection) and mind-wandering [14], and the central executive network, which is involved in working memory and goal-directed cognitive processes [15]. The seminal paper by Sridharan et al. [16], later replicated by Goulden et al. using a different methodology [17], evidenced the role of the SN in cognitive flexibility, facilitating the switch between DMN and CEN in response to internally and externally salient stimuli, respectively [8, 9, 13, 18]. The SN is also functionally connected to various subcortical and limbic structures such as the amygdala, thalamus, and striatum, which collectively contribute to the encoding of stimuli that are of high emotional, hedonic, and homeostatic salience [10, 19].

### Salience Network in the Processing of Physical and Socioemotional Pain

The processing of physical pain recruits a widespread collection of cortical and subcortical regions, known as the “pain matrix”, that largely overlaps with the SN [20]. The AI is postulated to be responsible for integrating sensory and interoceptive inputs to form subjective perceptions about the intensity and emotional aspects (i.e., unpleasantness) of pain. This information is transmitted to the ACC for evaluation of the saliency of the pain stimuli to inform attention allocation and decision-making [9, 21]. Meta-analysis shows robust activations of the insula and ACC in response to experimentally induced pain stimulation [2]. Instead of transient responses to the onset and offset of non-painful somatosensory stimuli, SN activation is prolonged throughout the delivery of painful stimuli [22]. Activation of SN regions is associated with the objective intensity of the pain stimuli [3] but is additionally modulated by varying cognitive processes and environmental factors to shape individuals’ perception of pain. The ability of the SN to integrate internal and external stimuli in processing pain allows individuals to prioritize attention to potential harm and execute adaptive responses [20], such as attributing more saliency to stimuli previously associated with harm or less to pain in the context of competing cognitive demands (i.e., fighting a war). Reducing expectation of pain intensity without changing the actual pain intensity attenuates neural responses to pain in SN regions (e.g., ACC, insula, and thalamus) as well as subjective ratings of pain intensity (i.e., the “placebo analgesia” effect) [23, 24]. Similarly, engaging in a cognitively demanding task that serves to distract subjects from pain stimulation reduces the neural response of the SN to pain stimulation [25], possibly by diverting saliency from the painful stimuli to the cognitive task.

Alterations in SN structure and function were reported in individuals with chronic pain. Meta-analysis of voxel-based morphometry data across chronic pain disorders indicates reduced gray matter volume within the SN, including the bilateral insula, cingulate cortex, and thalamus [26]. Meta-analysis of functional MRI data further shows that chronic pain elevates SN response to acute pain stimuli [27]. Functional connectivity within the SN also differs between healthy controls and individuals with chronic pain, but the directionality of change varies across studies, such that both enhanced [28, 29] and decreased [30] connectivity in chronic pain patients vs. controls have been reported. Nonetheless, aberrant SN functioning may contribute to impaired connectivity with other brain networks and have implications for altering pain processing. The activity of the SN is normally negatively correlated with that of the DMN [31], but individuals with chronic pain show increased SN-DMN functional connectivity (i.e., attenuated negative correlation) [32, 33]. These changes were associated with clinical pain symptomatology and pain sensitivity [29, 32] and may reflect deficits in cognitive flexibility that bias attention toward pain. Studies demonstrating increased SN functional connectivity with the attention and sensorimotor networks in pain patients compared to healthy controls [28, 33] further support the role of attentional focus to nociceptive afference in chronic pain.

Socioemotional pain encompasses feelings of distress stemming from social disconnectedness, rejection, disapproval, or loss. Research indicates similarities in the neural mechanisms involved in both physical and socioemotional pain. The mu-opioid system is implicated in both physical pain relief and social bonding [34]. Mu-opioid receptor agonists such as buprenorphine alleviate both the physical [35] and socioemotional pain [36], while mu-opioid receptor antagonist naloxone exacerbates them [37, 38]. The non-opioid pain reliever acetaminophen has also been found to alleviate socioemotional pain resulting from social rejection [39]. Clinically, pain disorders often coincide with psychological problems including feelings of social exclusion and loneliness, which in turn intensify physical pain [40, 41]. Addressing socioemotional pain and providing social support are crucial for the effective management of chronic physical pain [42]. Interestingly, there is a significant overlap between the neural substrates for physical and socioemotional pain. The ACC and AI are consistently activated during experiences of socioemotional pain [4, 5]. The AI is further involved in the processing of general physiological and psychosocial stress [43]. Protective factors such as self-esteem [44] and social support [45] are linked to reduced activity in these regions during socioemotional pain. Moreover, the ACC and AI facilitate the interplay between physical and socioemotional pain: physical pain reliever acetaminophen reduces SN brain response to social rejection [39], while social rejection heightens SN response to physical pain [46]. Together, these data highlight the role of the SN in the shared neural representation of physical and socioemotional pain.

### Salience Network Dysfunction in Substance Use Disorders

SUDs encompass a chronically relapsing cycle of binge/intoxication, withdrawal/negative affect, and preoccupation/anticipation that propels continual substance use despite negative consequences [47]. Studies examining the neurobiological underpinnings of SUDs traditionally focused on the reward pathways but have shifted toward recognizing the role of other brain networks [8, 48]. Specifically, structural and functional connectivity aberrations in the SN have been identified in individuals with SUDs. Across substances, patients with SUDs display lower gray matter volume in SN brain structures [6, 7, 49–52] and alterations in the connectivity pattern between the SN and other brain networks [53, 54]. Compared to healthy controls, individuals with opioid and cocaine use disorders demonstrate lower insula and ACC connectivity with brain regions of the CEN (e.g., dorsolateral prefrontal cortex, posterior parietal cortex) and the DMN (e.g., medial prefrontal cortex, posterior cingulate cortex) [55–57]. Additionally, opioid and alcohol use disorders are associated with increased connectivity between the ACC and regions of the reward circuitry, such as the nucleus accumbens and caudate nucleus, suggesting abnormal incentive salience processing [57–59]. Changes in functional connectivity within the SN have also been observed, with decreased within-network insula/ACC connectivity in cocaine use disorder [60] and increased connectivity in alcohol use disorder [61].

The impaired Response Inhibition and Salience Attribution (iRISA) model of addiction underscores SN neuroadaptations in perpetuating SUDs by heightening the saliency of substances of abuse at the expense of other non-drug-related processes, such as inhibitory control and natural reward processing [48]. In line with this model, meta-analyses of task-based functional MRI studies indicated drug cue-induced activation in SN regions in individuals with SUDs that is linked to increased drug craving [62–64]. Behavioral data further corroborated the model by demonstrating attentional biases toward drug vs. neutral cues across substances [65–68]. In cocaine use disorder, such attentional bias has been linked to increased functional connectivity among brain regions involved in salience attribution, including the bilateral frontoinsular cortex, dorsal ACC, and bilateral frontoparietal regions [66]. Likewise, attentional bias positively correlated with cue-induced brain activation in the insula and ACC in individuals with alcohol use disorder [68] and with ACC connectivity with the hippocampus in opioid use disorder [69].

In addition to contributing to biased salience attribution, SN aberrations in individuals with SUDs are also linked to impairments in cognitive control. Functional MRI studies (reviewed in [70]) demonstrated reduced activation of SN brain regions during cognitive control (e.g., measured by the Stroop and go/no-go paradigms) in smokers [71] and individuals with methamphetamine [72], cocaine [73], and opioid [74, 75] use disorders compared to healthy controls. Meta-analyses corroborated the findings of hypoactivity of the ACC [76] and insula [77, 78] across SUDs, though more research is warranted given potential publication bias and insufficient behavioral evidence [78]. Interestingly, a meta-analysis that focused on alcohol use disorder revealed increased rather than decreased ACC activation during inhibitory control in patients than healthy controls [79], suggesting potential discrepancy across substances and the need for future studies.

Additionally, SUDs are associated with increased risk propensity in decision-making [80] that may be attributable to increased ACC and reduced prefrontal activity [81, 82]. Several studies examining risky decision-making in individuals with SUDs reported less engagement of the insula during monetary gain and loss processing [83, 84]. ACC hypoactivity during decision-making was also reported in one meta-analysis of functional MRI data in individuals with alcohol use disorder [84]. Contrarily, individuals with cocaine use disorder exhibit increasing ACC activity when choosing riskier options, whereas ACC activity in control participants decreased with increasing risk-taking [85]. Gowin et al. also demonstrated increased ACC activation and lower insula activation during risky decision-making in individuals with methamphetamine use disorder compared to healthy controls [86]. In addition to functional MRI-measured SN activity during cognitive and decision tasks, structural deficits (e.g., reduced gray matter volume) and reduced connectivity within the SN have been implicated in poor executive performance [87, 88], increased impulsivity [89], and slower decision making in individuals with alcohol use disorder [90]. Similar associations were found between SN connectivity with other brain networks (e.g., DMN, CEN, reward circuitry) and increased behavioral impulsivity and executive dysfunction in alcohol [91, 92] and cocaine use disorders [93, 94].

### Salience Network at the Intersection of Pain and Substance Use Disorders

Chronic physical pain is highly comorbid with nicotine [95], alcohol [96], cannabis [97], opioid [98], and stimulant [99] use disorders [(see review [100]]. People struggling with chronic pain often turn to these substances as a way to cope [101]. In a cross-sectional analysis of individuals with illicit drug use in the last 3 months, 51% of individuals who used marijuana, cocaine, or heroin and 81% of individuals who misused prescription drugs reported having used drugs to self-medicate for physical pain [102]. More severe physical pain has been linked to a higher risk of relapse in patients being treated for opioid use disorder (OUD) [103]. Healthcare providers caring for patients with both pain and SUDs, especially OUD, face the challenge of weighing the benefits of prescribing opioids for pain relief against the risks of misuse, dependence, and diversion [104]. The task is further complicated by alterations in patients’ pain perception as a result of chronic exposure to opioids (e.g., opioid-induced hyperalgesia) [105, 106] and other drugs (nicotine [107, 108]; stimulants [109]; alcohol [110]), combined with impaired interoception [111] and poor self-awareness/insight [112, 113] that may hamper the accuracy in self-reported pain. In addition, patients grappling with SUDs are often confronted with various socioemotional difficulties, such as financial constraints [114], loneliness [115], interpersonal conflict [116], and experience of stigma and discrimination [117]. Such difficulties can lead to various consequences, including increased stress sensitivity [118], poor treatment adherence [119], and heightened risk of relapse [120]. The impact of socioemotional adversity can be particularly fatal in OUD, as socially isolated patients are more likely to die from opioid overdose [121]. The COVID-19 pandemic has further compounded these issues, with increased unemployment rates and social distancing measures exacerbating social adversities [122], contributing to a surge in opioid overdose deaths [123].

Altered SN function in SUDs may impair normal salience detection and result in aberrant responses to physical and socioemotional pain [8]. Among the limited MRI research that examined pain processing in SUDs patients, one study found that compared to healthy controls, patients with comorbid OUD and chronic pain had increased connectivity among regions of the SN and the reward circuitry during acute pain stimulation [124]. However, it is unclear to what extent the abnormal SN connectivity can be attributed to OUD or chronic pain. Another study showed a lack of the characteristic SN activation in response to physical and socioemotional pain in OUD patients, but direct comparison between OUD and control individuals did not reveal significant differences under stringent whole-brain correction for multiple comparisons [125]. The same team additionally found a negative correlation between insular gray matter volume and social pain in OUD patients [126]. Non-imaging studies on OUD revealed blunted subjective emotional reactions, heightened physiological responses, and deficient regulation in response to social rejection [127, 128], which was further associated with increased drug craving [128]. Lastly, we recently found preliminary evidence that family/social problems (e.g., abuse, interpersonal conflict, parental drug use, etc.) increased AI response to drug-related stimuli compared to natural reward stimuli in OUD patients [129]. It should also be noted that both SUDs and physical/socioemotional pain are closely related to various psychiatric problems (e.g., depression, anxiety, posttraumatic stress disorder) [130–132]. Functional and structural alterations in the SN are a recurring phenomenon across psychiatric disorders and may serve as a key mechanism for their comorbidity with SUDs and pain [133, 134]. Therefore, the identification of better treatment options for physical and socioemotional pain is critical and may have implications for preventing SUD, and vice versa.

The involvement of the SN in the processing of physical and socioemotional pain and its perturbations in SUDs underscore its potential as a target for treatment. Functional MRI-measured ACC and insula hyperactivity in response to drug cues and hypoactivity during cognitive tasks have been prospectively linked to treatment outcomes in patients with SUDs (e.g., relapse) [135–137]. Similar findings were obtained for the treatment of pain disorders, such that individual differences in the baseline connectivity and structural integrity of the SN were shown to predict treatment effectiveness [138, 139] and future recovery [140, 141]. Therefore, interventions targeting the SN may have therapeutic potential for treating SUDs. Real-time functional MRI neurofeedback is a technique that allows subjects to observe and modulate their own brain activity by viewing feedback provided in the form of a graphical “thermometer” of real-time neural responses in brain regions of interest. One study has shown the effectiveness of real-time functional MRI in helping smokers reduce cigarette cravings by volitionally reducing their ACC activity [142]. The method has also been examined in the intervention for alcohol use disorder, though with less success [143, 144]. In addition to real-time functional MRI, neuromodulation has been explored for its potential in SUD treatment [145, 146]. Non-invasive neuromodulation techniques include transcranial magnetic stimulation (TMS) and transcranial direct current stimulation (tDCS), which apply magnetic fields and low-intensity electrical currents, respectively, to the scalp to modulate regional neuronal activity [145]. While most TMS and tDCS research on SUDs have focused on the dorsolateral prefrontal cortex [145, 146], several studies suggest that TMS targeting the insula and ACC may reduce the craving for and/or the consumption of alcohol [147–149], tobacco [150–153], and cocaine [154] (but see [155]). Invasive neuromodulation such as deep brain stimulation has mostly focused on the effect of blocking neural transmission in the nucleus accumbens [146, 156]. Nevertheless, two studies showed the feasibility and effectiveness of ACC stimulation via implanted electrodes for reducing alcohol craving and consumption [157, 158].

Psychotherapies have also shown promise for improving SUD treatment outcomes, potentially by influencing the SN. Cognitive behavioral therapy (CBT) is a highly effective psychotherapeutic approach that teaches individuals to identify and counteract harmful thought patterns and environmental risk factors [159, 160]. One functional MRI study revealed decreased ACC engagement during cognitive control among individuals with SUDs following CBT, which might reflect reduced cognitive effort [161]. Another psychotherapeutic technique, mindfulness meditation, trains individuals to develop and maintain non-judgmental, non-overly reactive, and present-moment awareness of their thoughts and feelings. Mindfulness meditation has demonstrated efficacy in treating SUDs and related comorbidities (e.g., depression, anxiety) [162–165]. In healthy individuals, a month-long mindfulness meditation training increased SN functional connectivity with brain regions of the DMN and the CEN [166]. In individuals with OUD, mindfulness increased the correlation between the gray matter volume of the SN and that of the striatum and prefrontal cortex [167]. Lastly, among smokers, mindfulness increased ACC activity during resting state [168] and attenuated ACC activity and ACC-insula connectivity during exposure to smoking cues [169]. These findings demonstrated the SN’s involvement in psychotherapeutic interventions for SUDs, though more research is needed to further elucidate the mechanistic role of the SN.

## Conclusions and Future Directions

The review highlights the significance of the SN in mediating the interplay between SUDs and physical/socioemotional pain. It also identifies several critical gaps in knowledge that require future research. First, despite the emphasis of this review on the SN, it is important to recognize SUDs as a group of multifaceted neurocognitive disorders affecting a broad range of brain structures. The SN interacts with other brain regions, such as the prefrontal cortex and the limbic system [8–10, 13, 18, 19], that collectively contribute to the etiology and maintenance of SUDs [48, 170]. Similarly, both physical and socioemotional pain have profound effects on the brain that extend beyond the SN [2, 3, 171, 172]. Future studies should aim to deepen our understanding of the neurobiological mechanisms underlying the role of the SN in pain and SUDs. This includes exploring the SN’s interactions with other brain networks and their joint effects in shaping the experience of pain and the trajectories of substance use. Secondly, the role of the SN in SUDs may vary depending on the type of substance. For example, opioids are directly involved in the modulation of physical pain, and both the ACC and AI have high mu-opioid receptor availability [173]. In addition, consumption of substances such as nicotine [174] and cannabis [175] is heavily influenced by social context and potentially more closely related to socioemotional pain. A more fine-grained comparison across substances is warranted. Lastly, despite the significant advances in functional MRI research, the technique is not without its limitations. The spatial and temporal resolutions of functional MRI are insufficient for a precise understanding of neuronal processes. Additionally, whether functional MRI data can reliably predict clinical outcomes remains a subject of debate [176, 177]. Future work that integrates neuroimaging discoveries with other preclinical and clinical studies will not only promise a more holistic and precise delineation of the SN’s involvement in pain and SUDs but also pave the way for the development of next-generation treatments.

## Data Availability

No datasets were generated or analysed during the current study.
